# Temporal Change of the Content of 10 Oligosaccharides in the Milk of Chinese Urban Mothers

**DOI:** 10.3390/nu8060346

**Published:** 2016-06-08

**Authors:** Sean Austin, Carlos A. De Castro, Thierry Bénet, Yangfeng Hou, Henan Sun, Sagar K. Thakkar, Gerard Vinyes-Pares, Yumei Zhang, Peiyu Wang

**Affiliations:** 1 Nestlé Research Centre, Vers-Chez-Les-Blanc, Lausanne 1000, Switzerland; CarlosAntonio.DeCastro@rdls.nestle.com (C.A.D.C.); thierry.benet@rdls.nestle.com (T.B.); Sagar.Thakkar@rdls.nestle.com (S.K.T.); 2 Nestlé Research Center Beijing, Beijing 100095, China; Yangfeng.Hou@rd.nestle.com (Y.H.); shn1984@126.com (H.S.); Gerard.VinyesPares@RD.nestle.com (G.V.-P.); 3 Department of Nutrition and Food Hygiene, School of Public Health, Peking University Health Science Center, Beijing 100191, China; zhangyumei@hsc.pku.edu.cn; 4 Department of Social Medicine and Health Education, School of Public Health, Peking University Health Science Center, Beijing 100191, China; wpeiyu@bjmu.edu.cn

**Keywords:** human milk oligosaccharides, lactation, HPLC, sialyllactose, fucosyllactose, lacto-*N*-tetraose, lacto-*N*-neotetraose, lacto-*N*-fucosylpentaose

## Abstract

Breastfed infants tend to be less prone to infections and may have improved cognitive benefits compared to formula-fed infants. Human milk oligosaccharides (HMO) are the third most abundant component of human milk, but are absent from formulae. They may be partially responsible for the benefits of breastfeeding. In this cross-sectional observational study, the HMO composition of milk from Chinese mothers was studied to determine the impact of stage of lactation, mode of delivery and geographical location. The content of 10 HMO was measured by HPLC in 446 milk samples from mothers living in three different cities in China. Around 21% of the samples contained levels of 2′-fucosyllactose (2′-FL) below the limit of quantification, which is similar to the frequency of fucosyltransferase-2 non-secretors in other populations, but 2′-FL was detected in all samples. Levels of most of the HMO studied decreased during the course of lactation, but the level of 3-fucosyllactose increased. Levels of 2′-FL and 3-fucosyllactose seem to be strongly correlated, suggesting some sort of mechanism for co-regulation. Levels of 6′-sialyllactose were higher than those of 3′-sialyllactose at early stages of lactation, but beyond 2–4 months, 3′-sialyllactose was predominant. Neither mode of delivery nor geographical location had any impact on HMO composition.

## 1. Introduction

Exclusive breastfeeding is considered to be the best nutrition for the new-born infant up to the age of six months, and it is recommended to continue supplementing the diet with breast milk for one year or longer [1]. Compared to bottle-fed infants, breastfed infants are less prone to infections [2] and may have improved cognitive development [3]. Breastfeeding has also been linked to a reduction in the risk of developing childhood leukemia [4].

To understand why breastmilk provides so many benefits, it is necessary to have a complete understanding of breast milk composition. One of the biggest differences between breastmilk and infant formula is in the non-lactose oligosaccharide (NLO) content. At concentrations of 20–25 g/L in colostrum and 10–15 g/L in mature milk, these oligosaccharides, normally termed human milk oligosaccharides (HMO), are the third most abundant solid component of human milk after lactose and fat [5,6,7]. In bovine milk, the NLO content, at around 0.1 g/L [8,9], is almost negligible. Likewise, many infant formulae contain negligible amounts of NLOs, although manufacturers have started to add non-digestible carbohydrates, such as inulin, fructooligosaccharides, polydextrose and galacto-oligosaccharides, in attempts to mimic some of the postulated benefits of the HMO.

The HMO are built on a series of 13 core structures. The simplest core structure is lactose, which can be elongated by the additions of the disaccharides β-d-galactosyl-(1→4)-β-d-glucosamine or β-d-galactosyl-(1→3)-β-d-glucosamine through β-1,3- or β-1,6-linkages to form the others [5]. The core structures themselves can be further decorated by the additions of *N*-acetylneuraminic acid residues linked via α-2,3- or α-2,6-linkages to form the series of acidic HMO or by fucose residues linked via α-1,2-, α-1,3- or α-1,4-linkages to form the series of neutral fucosylated oligosaccharides. Some of the core structures remain in the milk without further modifications, and others are decorated by both fucose and *N*-acetylneuraminic acid residues. Although it is predicted that over 200 HMO probably exist [5], only a little over 100 have been fully characterized [10,11,12].

The relative abundance of the various HMO is determined by the expression and activity of a number of glycosyltransferase enzymes. Of particular importance are the fucosyltransferases, which are encoded for by the secretor (Se) and Lewis (Le) genes. The different expression levels of the encoded enzymes gives rise to four main milk groups [13]. The most common group is synthesized by mothers who are positive for both secretor and Lewis genes (Se+, Le+). They synthesize a large number of different fucosylated oligosaccharides, including 2′-fucosyallactose (2′-FL), 3-fucosyallactose (3-FL), lacto-*N*-fucosylpentaose-I (LNFP-I), LNFP-II and LNFP-III, because they express all of the enzymes required to attach fucose residues through α-1,2-, α-1,3- and α-1,4-linkages. Milk synthesized by mothers who are secretor positive, Lewis negative (Se+, Le−), contains oligosaccharides, such as 2′-FL, 3-FL, LNFP-I and LNFP-III, but structures containing α-1,4-linked fucose residues, such as LNFP-II, are absent. Mothers who are secretor negative, Lewis positive (Se−, Le+), lack structures containing α-1,2-linked fucose, such as 2′-FL and LNFP-I, in their milk, but the milk contains structures, such as 3-FL, LNFP-II and LNFP-III. Mothers who are negative for both secretor and Lewis genes are unable to produce HMO containing α-1,2- or α-1,4-linked fucose; thus, structures such as 2′-FL, LNFP-I and LNFP-II are all missing, and the only fucosylated structures in the milk are those containing solely the α-1,3-linkage, such as 3-FL, LNFP-III and LNFP-V. Although the milk of most individuals can be classified into one of these four groups, there remains significant variation of individual oligosaccharide levels within the groups, and less common oligosaccharide profiles exist. For example, Prieto [14] reported that in a study of milk from 435 mothers, two individuals had milk profiles that did not fit in to any of the four groups. The two individuals lacked oligosaccharides containing α-1,3-linked fucose residues, in particular 3-FL. It is also known that the secretor and Lewis genes can contain defect mutations resulting in the generation of enzymes with poor activities. For example, a third secretor phenotype is known to exist in some Asian populations; the weak secretor [15]. Weak secretors produce fucosyltransferase-II (FucT2), the enzyme responsible for synthesizing structures containing α-1,2-linked fucose residues, but due to modifications in the amino acid sequence, the activity of the enzyme is significantly reduced; thus, although oligosaccharides, such as 2′-FL and LNFP-I, are present in the milk, their concentrations are below those typically found in the milk of secretor mothers.

Since the HMO content of human milk is so important, it is logical to expect that it may be responsible for at least some of the health benefits experienced by breastfed infants. It is postulated that the HMO act through several different mechanisms to protect the infant from infection: (1) by acting as a preferred substrate for the growth of selected “good” bacteria in the gastrointestinal tract; (2) by acting as decoy molecules that are bound by pathogenic bacteria, preventing the bacteria from binding to the surface of the host cells; and (3) by modulating the immune system through direct interactions.

The profile of a healthy gastrointestinal microbiome has not really been established, although one would expect that it looks similar to that of a breastfed infant. Promotion of the growth of “good” bacteria is postulated to result in decreased colonic pH, which in turn inhibits the growth of pathogenic bacteria. In addition, a strong population of “good” bacteria may out-compete the pathogenic bacteria, and thus, the infant has reduced risk of gastrointestinal infection [16,17]. It has further been postulated that complex interactions between HMO, the gut microbiota and host cells further cooperate to modulate inflammation and infections [18]. Albrecht [19] and Coppa [20] compared the oligosaccharide composition of breast milk and of the feces of the babies fed that milk. They demonstrated that the amount of oligosaccharides present in the feces was only about 40%–50% of that ingested, demonstrating that the HMO can be used as a carbon source for the growth of the gastrointestinal microbiota. They also noticed that some specific HMO were preferred over others. Asakuma [21] has shown that certain strains of Bifidobacteria contain the enzymatic machinery capable of utilizing oligosaccharide structures containing features specific to HMO and that to process the HMO, several species must work in concert. The profiles of the gastrointestinal microbiota between breastfed and formula-fed infants are thus different [22,23,24], and recent data suggest that the microbiota profile of breastfed infants varies depending on the breast milk oligosaccharide pattern [25].

The HMO structures strongly resemble the non-reducing termini of glycans present on the surface of human cells. Infection by pathogens often involves, as the first stage, the binding of the pathogen to the cell surface through interactions between lectins and glycans. Since the HMO closely resemble the cell surface glycans, it is possible that HMO are recognized by the pathogen lectins, and thus, the pathogens are inhibited from binding to host epithelial cells [26]. Various *in vitro* experiments and experiments using animal models have demonstrated that some HMO may prevent infection by various pathogens through such a mechanism [27,28,29,30,31,32,33].

The HMO may also directly interact with the host’s own cells. Angeloni [34] demonstrated that the glycans expressed on the surface of CaCo 2 cells were modulated when exposed to 3′-SL. HMO have been detected in the urine [35] and plasma [35,36] of breastfed infants at levels sufficient to suggest that they may have some physiological impact. *In vitro* experiments have shown that the oligosaccharides can affect cell-cell adhesion and may play some immunomodulatory roles and regulate inflammatory responses [16,17,37,38].

It has been suggested that milk may be a carrier for glyconutrients, in particular sialic acids [39]. Humans are able to synthesize sialic acid; however, it has been speculated that this synthesis is insufficient in the neonate, and thus, it requires additional sialic acid from the diet [40]. Sialic acid is present in high concentrations in the central nervous system and in brain grey matter, usually as a component of gangliosides [3]. Breastfed infants receive a higher amount of sialic acid in their milk than formula-fed infants [41], and breastfed infants tend to perform better in cognitive tests compared to formula-fed infants. Taken together, the evidence suggests that dietary sialic acid may be important for infant brain development [3]. Wang [42] tested this hypothesis in piglets and demonstrated that supplementing their diet with additional sialic acid improved their cognitive performance. Sialic acid in breast milk is predominantly oligosaccharide bound [41]. Concentrations of total sialic acid in colostrum were reported to be around 5 nmol/L, which drops to around 2 nmol/L after one month and to 1 nmol/L after three months [41]; at all stages, 70%–75% of the total sialic acid was oligosaccharide bound.

Although it is known that more than 100 HMO exist in human milk, quantitative data are only available for around 30 [16,43,44,45,46,47,48,49,50,51,52,53,54,55,56,57,58,59,60,61,62,63], but those 30 represent a significant proportion of the total HMO mass. Most of the quantitative data collected to date are based on analysis by high performance liquid chromatography (HPLC), either with pulsed amperometric detection (PAD) of the non-derivatized oligosaccharides [16,43,44,45,46,47,48,49,50] or with UV or fluorescence detection of suitably-derivatized oligosaccharides [51,52,53,54,55,56,57,58]. Alternative methods have also been explored, such as HPLC coupled with mass spectrometry (HPLC-MS) [59,60], capillary electrophoresis (CE) [54,62] and nuclear magnetic resonance spectroscopy (NMR) [64].

Galeotti and co-workers [61] compared the results of HMO analysis using three different techniques (HPLC-MS, HPLC-PAD, CE-UV). For lactose measurement, the three techniques were in reasonable agreement; however, for the individual HMO, the situation was different, with the three techniques generally giving quite different results. For example, when difucosyllacto-*N*-hexaose was measured in milk from a (Se+Le+) donor, the result from HPLC-PAD was 3.8 g/L, from HPLC-MS 2.8 g/L and from CE-UV 1.2 g/L. In other cases, two techniques would give similar results, while the third was different; for example, LNnT levels measured in the milk of the same donor were 2.2 g/L by HPLC-PAD, 3.7 g/L by HPLC-MS and 4.1 g/L by CE-UV. There were no clear trends in the data to suggest that one technique or another had specific issues, *i.e.*, it was not always the same two techniques that gave similar results, nor was it always the same technique giving the highest or lowest results. This observation raises some questions about the appropriateness of methods and certainly suggests that methods should be thoroughly validated before applying them to samples from large cohorts. Different methodologies require different sample preparation protocols, and different labs using the same final analytical technique will often apply different procedures for sample preparation. Sample preparation probably plays a large part in the variability observed between the methods. The purity of commercial HMO purchased as standards can also vary between batches and suppliers, as can the methods used by the suppliers to assess purity. This can also have an important impact on the final results. Since the data reported by Galeotti and co-workers [61] were published over a number of years [47,61,65], one can imagine that the standards employed for calibration may have come from different sources (or batches), and this may also be a contributing factor to the variability observed.

Several studies have characterized the types and levels of some oligosaccharides during lactation [16,43,44,45,47,48,49,51,52,54,55,57,59,60]; however, most of them have focused on the first few weeks of lactation, and there are only a few that have explored the oligosaccharide composition beyond one or two months [43,44,48,55,57]. In addition, to date, there is little data available characterizing the oligosaccharide composition of Chinese mother’s milk [62]. This study hopes to address some of those gaps.

The aim of the work reported here was to collect data on the HMO composition of milk from mothers living in mainland China and to assess the impact of stage of lactation, geographical location within China and mode of delivery, since China has one of the highest rates of caesarean section birth in the world [66]. The work reported here is part of the larger initiative Maternal Infant Nutrition Growth (MING) study, conducted in a large cohort of urban Chinese mothers [67].

## 2. Materials and Methods

### 2.1. Trial Design

This work was part of MING study, a cross-sectional study designed to investigate the dietary and nutritional status of pregnant women, lactating mothers and young children aged from birth up to three years living in urban areas of China. In addition, the human milk composition of Chinese lactating mothers was characterized. The study was conducted between October 2011 and February 2012. A multi-stage milk sampling from lactating mothers in three cities (Beijing, Suzhou and Guangzhou) was performed for breast milk characterization. In each city, two hospitals with maternal and child care units were randomly selected, and at each site, mothers at lactation period 0–240 days were randomly selected based on child registration information. Subjects included in the period 0–5 days were recruited at the hospital, whereas all other subjects were requested by phone to join the study; if participation was dismissed, a replacement was made. The response rate was 52%. Recruitment and milk sampling, as well as baseline data collection were done on separate days.

A stratified milk sampling of 540 lactating mothers in six lactation periods of 0–4, 5–11 and 12–30 days and of 1–2, 2–4 and 4–8 months was obtained in the MING study. Only 446 milk samples were included in the HMO study, as the 0–4 days stage could not be included due to the limited volume of milk collected in this period.

Eligibility criteria included women between 18 and 45 years of age giving birth to a single, healthy, full-term infant and exclusive breastfeeding at least until 4 months. Exclusion criteria included gestational diabetes, hypertension, cardiac diseases, acute communicable diseases and postpartum depression. Lactating women who had nipple or lacteal gland diseases, who had been receiving hormonal therapy during the three months preceding recruitment or who had insufficient skills to understand study questionnaires were also excluded.

All subjects completed a general questionnaire including socio-economic and lifestyle aspects of the mother. Self-reported weight at pre-pregnancy and at delivery, number of gestational weeks at delivery and delivery method were also recorded. Additionally, a physical examination evaluated basic anthropometric parameters (height, weight, mid-arm circumference) blood pressure and hemoglobin.

Data collection was done through face-to-face interviews on the day of human milk sample collection. In addition, date of birth and gender information of the baby were collected after the data collection since the data were not included in the initial questionnaires. Subjects were contacted by phone and were asked to clarify these two aspects retrospectively.

### 2.2. Milk Collection and Storage

Breast milk sampling was standardized for all subjects, and an electric pump (Horigen HNR/X-2108ZB, Xinhe Electrical Apparatuses Co. Ltd.) was used to sample the milk. Samples were collected at the second feeding in the morning (9:00–11:00 a.m.) to avoid circadian influence on the outcomes. A single full breast was emptied, and an aliquot of 40 mL was secured for characterization purposes. The rest of the milk was returned to the mother for feeding to the infant. Each sample was distributed in 1-mL freezing tubes, labeled with the subject number, stored at −80 °C and analyzed within 6 months of collection.

### 2.3. Ethical and Legal Considerations

The study was conducted according to the guidelines in the Declaration of Helsinki. All of the procedures involving human subjects were approved by the Medical Ethics Research Board of Peking University (No. IRB00001052-11042). Written informed consent was obtained from all subjects participating in the study. The study was also registered on ClinicalTrials.gov with the number identifier NCT01971671.

### 2.4. Analytical Method

#### 2.4.1. Chemicals and Reagents

All chemicals were sourced from Sigma or Merck (Darmstadt, Germany), unless otherwise stated. Water was deionized to 18 MΩ by a Milli-Q system (Merck-Millipore, Darmstadt, Germany). All HMO standards were purchased from Elicityl (Crolles, France) with purity assessed by quantitative NMR.

#### 2.4.2. Sample Preparation

Homogenized milk samples (~40 mg) were accurately weighed into microtubes and mixed with a solution of laminaritriose (3000 µmol/L, 20 µL). An aliquot (20 µL) was transferred to a safe-lock microtube, and the labelling solution was added (2-aminobenzamide (0.35 mol/L) + sodium cyanoborohydride (1.0 mol/L) in dimethylsulfoxide containing acetic acid (30%), 200 µL). The solution was mixed well, and the closed tubes were placed in a water bath at 65 °C for 2 h. The tubes were then cooled at 4 °C for 10 min, and a mixture of acetonitrile/water (75/25 *v*/*v*, 1.5 mL) was added. This solution was then transferred to vials suitable for the autosampler of the liquid chromatograph. The labelled oligosaccharides were separated and quantified by UHPLC with fluorescence detection using a Thermo Ultimate 3000 RS UHPLC system (Thermo Scientific, Waltham, MA, USA) comprised of two high pressure mixing gradient pumps (one used as the loading pump and the other as the analytical pump), an autosampler maintained at 10 °C, a column compartment maintained at 60 °C and a fluorescence detector operating with an excitation wavelength of 330 nm and emission wavelength of 420 nm. The system was equipped with a 2-position 10-port switching valve located outside the column compartment; this was plumbed in a configuration suitable for on-line removal of the excess labelling reagent using a BEH Amide pre-column (Waters, Milford, MA, USA), as previously described [68]. The analytical column was a Waters BEH Amide (1.7 µm, 3.0 mm × 150 mm), and the oligosaccharides were eluted with a gradient of acetonitrile (Eluent A) and ammonium formate solution (50 mmol/L, pH 4.5, Eluent B) at 0.8 mL/min as follows: loading pump: 0 min, 95% A; 2.5 min, 95% A (switch valve to divert the sample on the analytical column); 4.0 min, 10% A; 6.0 min, 10% A (stop flow); 62 min, 10% A (start flow); 65 min 95% A; 66 min, 95% A (switch valve); 70 min, 95% A; analytical pump: 0 min, 90% A; 6.0 min, 90% A; 40 min, 82% A; 60 min, 80.5% A; 61 min, 30% A; 65 min, 30% A; 66 min, 90% A; 70 min 90% A.

### 2.5. Method Validation

The method was validated for the determination of 10 different HMO in human milk. For each oligosaccharide, the linear response of the detector was checked over the concentration range expected to be present in human milk samples. Each oligosaccharide was analyzed at nine different levels in triplicate. A linear regression was performed, and linearity was assessed from the square of the correlation coefficient (*r*^2^) and the plot of residuals.

To determine the trueness and precision of the method, a milk sample was selected and spiked with the oligosaccharide standards at 3 levels (the levels were adapted for each oligosaccharide to cover the concentration range expected in milk). The non-spiked sample and the spiked samples were analyzed in duplicate on 6 different days (total of 12 individual analyses). The spike recovery (difference between spiked sample and blank) was used to determine trueness; data from the duplicate analyses were used to determine repeatability (RSD(r)); and data from the between day analyses were used to determine intermediate reproducibility (RSD(iR)).

### 2.6. Data Analysis

Prior to statistical analysis, the data were treated as follows: (1) all concentrations that were below the detection limit of the method were set to 0 (zero); (2) all concentrations above the detection limit, but below the limit of quantification were set to a level of half the LoQ. For example, if the LoQ for the analyte was 100 mg/kg and the measured amount was 88 mg/kg, then the level was set to 50 mg/kg.

A multiple regression model to explain the HMO concentration was applied. The distribution of the residuals was checked via the Box-Cox transformation method, and a logarithmic transformation was shown to be adequate for all HMO. Prior to logarithmic transformation, a small increment (1 × 10^−10^ mg/kg) was added to the HMO concentration to enable transformation of eventual zero values. The following model was used:

log(concentration) = timeframe + sex + delivery + city + ε
(1)

The above model was the general model that was used to test for the effect of the stage of lactation (timeframe) on oligosaccharide concentration taking into consideration other variables, such as gender, mode of delivery (natural *vs.* cesarean) and geographic location (city). The term ε refers to a residual error (calculated as the observed value minus the predicted value). With this model, contrast estimates were calculated comparing the successive timeframes (5–11 days *vs.* 12–30 days, 12–30 days *vs.* 1–2 months, *etc.*) to observe at which timeframes there were significant changes in HMO concentration.

Similar models were used to assess the impact of the other variables with the difference of taking into account the interaction effect of time with the variable in question. For example, the following model was used so that a comparison of the geographical location (city) can be made for each timeframe:

log(concentration) = timeframe × city + sex + delivery + ε
(2)

The same principle was applied in looking at differences for the mode of delivery. Statistical analyses were performed both on the transformed dataset (as described) and on the dataset with data below LoQ being removed.

## 3. Results

### 3.1. Study Population

In this cross-sectional study, milk was collected from 450 mothers, 90 for each of the lactation stages defined in the study, living in three different cities (Beijing, Guangzhou and Suzhou). The mothers were predominantly non-smokers with a mean age of 27 years. The incidence of cesarean delivery varied from 38% to 59% between the groups. The complete study population characteristics are listed in Table 1. Although 450 samples were collected, only 446 were analyzed for HMO because two samples from the 5 to 11 days group and two samples from the 12 to 30 days group had insufficient volume for all of the analyses included in the larger study.

### 3.2. Analytical Method Performance

**Figure 1:**
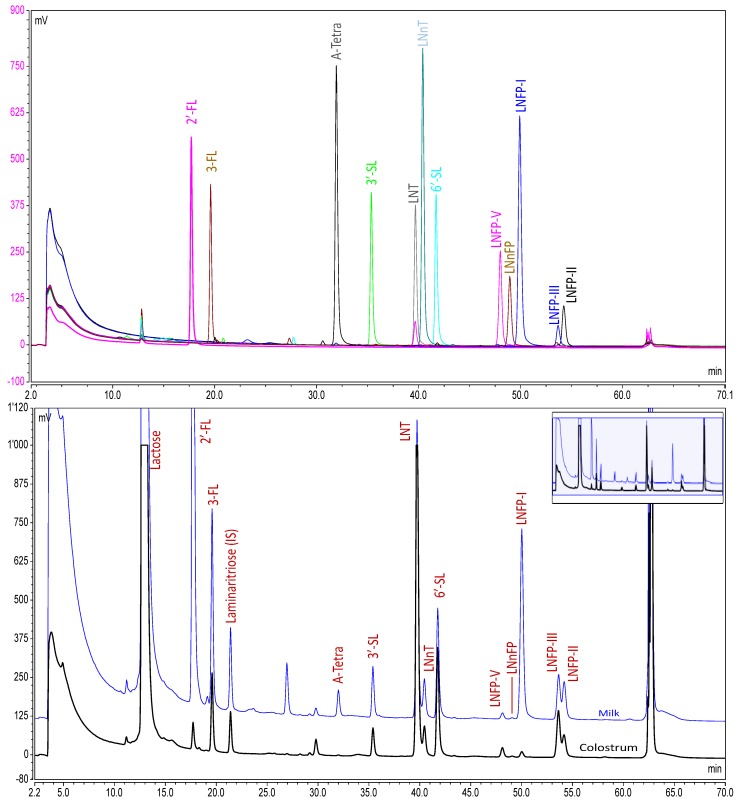
Chromatograms of human milk oligosaccharides (HMO). Top panel: overlay of the chromatograms of HMO standards injected individually. Lower panel: overlay of the chromatograms of pooled milk and pooled colostrum (both purchased from Lee Biosolutions, Maryland Heights, MO, USA).

An UHPLC-FLD method has been developed that is capable of the separation of at least 12 oligosaccharides with a run time of 1 h (Figure 1). The identity of the HMO were established by checking the retention times against those of known standards. LC-MS experiments were performed during method development to ensure that other oligosaccharides did not co-elute with any of the HMO being analyzed. Although 12 oligosaccharides were separated and identified in the chromatogram, at the time of analysis, only 10 were available as high quality quantitative standards (defined as available in quantities of 100 mg or above and supplied with accurately-known purity assessed by quantitative NMR). Therefore, the quantitative analysis was only performed on those 10.

The method has been validated (Table 2) and has excellent recovery for all oligosaccharides (98.7%–105%); the precision is also good, with relative standard deviations under repeatability conditions (RSD(r)) between 1.6% and 8.1% and relative standard deviations under intermediate reproducibility conditions (RSD(iR)) between 2.6% and 8.1%. The performance was considered sufficient for the analysis of the 446 milk samples coming from the clinical trial.

### 3.3. Analysis of Milk Samples

A total of 446 milk samples were analyzed, 88 from 8 to 11 days post-partum, 88 from 12 to 30 days post-partum, 90 from 1 to 2 months post-partum, 90 from 2 to 4 months post-partum and 90 from 4 to 8 months post-partum.

The majority of samples contained levels of each HMO above the LoD of the method (Table 3). The exception to this was A-tetrasaccharide, which was detected in only 65 out of the 446 samples analyzed (*i.e.*, 15% of the samples). Significant numbers of samples contained levels of 2′-fucosyllactose (2′-FL), A-tetrasaccharide, lacto-*N*-fucosylpentaose-I (LNFP-I), lacto-*N*-fucosylpentaose-V (LNFP-V) and lacto-*N*-neofucosylpentaose (LNnFP) below the LoQ of the method (Table 4).

Of the parameters investigated (stage of lactation, mode of delivery and geographic location within China), only the stage of lactation had a significant impact on the concentration of any of the oligosaccharides studied.

2′-FL was detected in all samples, although in 96 samples (representing 22% of all samples), the levels were below the method LoQ (53 mg/kg). Measurable levels of 2′FL ranged from 56 mg/kg to 4900 mg/kg with mean and median concentrations at early stages of lactation being higher than those at later stages (Table 3 and Table 4). Mean concentrations of 2′FL were only statistically significantly different from earlier stages at 2–4 months and 4–8 months of lactation (and only when data below LoQ were eliminated from the dataset (Table 4). Nevertheless, there is a trend for the concentration to decrease throughout all of the stages from 5–11 days to 4–8 months.

3-FL was detected in all samples; only 10 samples (representing 2% of all samples) contained levels of 3-FL below the method LoQ (43 mg/kg). Measurable levels of 3-FL ranged from 47 mg/kg to 5900 mg/kg with mean and median concentrations at later stages of lactation being higher than those at earlier stages (Table 3 and Table 4). Mean levels of 3-FL were statistically significantly different from the preceding stage of lactation at 12–30 days and at 2–4 months. The mean concentrations at all later stages of lactation were statistically significantly different from those at 5 to 11 days (Table 3 and Table 4).

3′-SL was detected in all samples at levels above the method LoQ (23 mg/kg) ranging from 43 mg/kg to 260 mg/kg with mean and median concentrations at earlier stages of lactation being higher than those at later stages (Table 3 and Table 4). Mean levels of 3′-SL were statistically significantly different from the preceding stage of lactation at 12–30 days and at 1–2 months. The mean concentrations at all later stages of lactation were statistically significantly different from those at 5 to 11 days (Table 3 and Table 4).

6′-SL was detected in all samples; only 20 samples (representing 4% of all samples) contained levels of 6′-SL below the method LoQ (22 mg/kg). Measurable levels of 6′-SL ranged from 22 mg/kg to 690 mg/kg with mean and median concentrations at earlier stages of lactation being higher than those at later stages (Table 3 and Table 4). Mean levels of 6′-SL were statistically significantly different from the preceding stage of lactation at all stages after 5–11 days (Table 3 and Table 4).

LNT was detected in all samples, at levels above the method LoQ (14 mg/kg) ranging from 21 mg/kg to 3000 mg/kg with mean and median concentrations at earlier stages of lactation being higher than those at later stages (Table 3 and Table 4). Mean levels of LNT were statistically significantly different from the preceding stage of lactation at 12–30 days, 1–2 months and 2–4 months (Table 3 and Table 4).

LNnT was detected in all samples; only 34 samples (representing 8% of all samples) contained levels of LNnT below the method LoQ (20 mg/kg). Measurable levels of LNnT ranged from 20 mg/kg to 390 mg/kg with mean and median concentrations at earlier stages of lactation being higher than those at later stages (Table 3 and Table 4). LNnT concentrations were statistically significantly different from the preceding stage of lactation at all stages after 5–11 days (Table 3 and Table 4).

LNFP-I was detected in 382 samples (representing 86% of all samples); 95 samples (representing 21% of all samples) contained levels of LNFP-I below the method LoQ (15 mg/kg). Measurable levels of LNFP-I ranged from 15 mg/kg to 4000 mg/kg with mean and median concentrations at earlier stages of lactation being higher than those at later stages (Table 3 and Table 4). Mean levels of LNFP-I were statistically significantly different from the preceding stage of lactation only at 2–4 months (Table 3 and Table 4). Mean levels of LNFP-I at 1–2 months, 2–4 months and 4–8 months were all significantly different from the mean level at 5 to 11 days when concentrations below LoQ were assigned a level of half LoQ (Table 3). When LNFP-I levels below LoQ were eliminated from the dataset, mean levels of LNFP-I were statistically significantly different from the mean level at 5–11 days only at 2–4 months and 4–8 months (Table 4).

LNFP-V was detected in 431 samples (representing 97% of all samples); 161 samples (representing 36% of all samples) contained levels of LNFP-V below the method LoQ (13 mg/kg). Measurable levels of LNFP-V ranged from 13 mg/kg to 250 mg/kg with mean and median concentrations at earlier stages of lactation being higher than those at later stages (Table 3 and Table 4). Mean levels of LNFP-V were statistically significantly different from the preceding stage of lactation only at 1–2 months, although mean levels at 1–2 months and 4–8 months were significantly different from the mean level at 5 to 11 days (Table 3 and Table 4).

LNnFP was detected in 398 samples (representing 89% of all samples); 367 samples (representing 82% of all samples) contained levels of LNnFP below the method LoQ (12 mg/kg). Measurable levels of LNnFP ranged from 12 mg/kg to 51 mg/kg. Mean and median concentrations at 5–11 days appear to be slightly higher than those at later stages (Table 3 and Table 4). When LNnFP concentrations below LoQ were assigned a level of half LoQ (*i.e.*, 6.2 mg/kg), the mean level of LNnFP was statistically significantly different from the preceding stage of lactation only at 12–30 days, although mean levels at 12–30 days, 1–2 months, 2–4 months and 4–8 months were significantly different from the mean level at 5 to 11 days (Table 3). When LNnFP levels below LoQ were eliminated from the dataset, mean levels of LNnFP were statistically significantly different from the preceding stage of lactation only at 12–30 days, but mean levels at 12–30 days and 4–8 months were statistically significantly different from the mean level at 5 to 11 days (Table 4).

## 4. Discussion

### 4.1. Factors Influencing Oligosaccharide Expression

Of the factors investigated, only stage of lactation appeared to have any influence on the oligosaccharide composition of the milk. No significant correlations between oligosaccharide concentration and geographical location or mode of delivery were found.

Radzanowski [63], Musumeci [49] and Erney [43] previously investigated the influence of geographical location on milk oligosaccharide composition. All studies found significant differences in oligosaccharide profiles between different geographical locations. However, the different geographical locations in the previous studies included different countries and continents. Erney [43] attributed the different oligosaccharide profiles between regions to evolutionary-driven genetic differences between the inhabitants of the different countries. Although China is a large country, the majority of the populations in the cities involved in this study are of Han origin (>90% of recruited subjects) and, thus, may be too genetically similar to manifest significant differences in milk oligosaccharide composition. Nevertheless, within China, there is still scope to further diverge the locations for recruitment of volunteers and to increase the ethnic diversity of the recruited subjects. It would thus be possible that regional differences could exist within the country.

It is well established that the oligosaccharide composition varies with lactation stage [16,43,44,45,47,48,49,51,52,54,55,57,59,60]; however, most studies have focused on the first few weeks of lactation; only a few have explored the oligosaccharide composition beyond one or two months [43,44,48,55,57]. To the best of our knowledge, this is the first large study characterizing the change of the HMO concentration during lactation for a population of the Chinese mainland, and it is the first time that A-tetrasaccharide and LNnFP have been included as analytes in such a study. For seven of the oligosaccharides (2′-FL, 3′-SL, 6′-SL, LNT, LNnT, LNFP-I, LNFP-V), the concentration started high during early stages of lactation then reduced with progression to later stages. This is in general agreement with trends already observed in the literature [43,44,48,55,57] in other populations. 3-FL was unique in this dataset, being the only oligosaccharide that increased during lactation. This is also in agreement with previously-observed trends [43,44,48,55,57]. Due to the low concentrations of LNnFP and the rarity of A-tetrasaccharide, the dataset for these oligosaccharides is limited, and no clear trends were observed over the course of lactation.

### 4.2. Fucosylated Oligosaccharides

It is known that levels of fucosylated oligosaccharides are controlled (at least in part) by fucosyltransferase enzymes coded for by the secretor gene and the Lewis blood group genes [14,15,43]. It has been shown that the presence of α-1,2-linked fucosylated oligosaccharides in the milk protects the infant against diarrhea [69,70] and a range of other pathogenic microorganisms [33].

A-tetrasaccharide has only been reported in human milk in one other study [71], which reported that it would only be present in the milk of mothers that were of Blood Group A and who expressed the secretor gene. Expression of the secretor gene can be predicted from the presence of 2′-FL and LNFP-I in the milk [50]. In this study 21% of samples had levels of 2′-FL and LNFP-I below the method LoQ (Table 4), which is in line with the expected fraction of the population who do not express a fully–functioning FucT2 enzyme [72]. In China, approximately 23%–27% of the population is Blood Group A [73]. Therefore, according to Kobata’s predications, we may expect 18%–22% of the samples to contain A-tetrasaccharide, which is close to the 15% observed. Only 16 samples contained levels of A-tetrasaccharide above the LoQ of our method (Table 4); the concentrations varied from 20 to 160 mg/kg, which is higher than the 0.2–13 nmol/mL (*i.e.*, 0.14–9.0 mg/L) previously reported by Kobata [71]; however, the other 50 samples in which A-tetrasaccharide was detected should have levels between 4.9 and 20 mg/kg (*i.e.*, between the method LoD and LoQ), and that range overlaps with that reported previously.

2′-FL and LNFP-I are two of the oligosaccharide products resulting from the activity of the FucT2 enzyme. Although 21% of samples had levels of 2′-FL and LNFP-I below the method LoQ (similar to the general incidence of non-secretors [72]), 2′-FL was detected in every sample analyzed. We confirmed that the signal detected in such samples was 2′-FL via additional LC-MS/MS experiments on selected samples to confirm that the peak identified as 2′-FL had the appropriate mass and fragmentation pattern. This suggests that in the Chinese population, the FucT2 enzyme may never be completely knocked-out or there is an alternative route for synthesizing 2′-FL that is as yet unknown.

3-FL was detected in all milk samples; however, a small number (2.2%) contained levels of 3-FL below the LoQ (43 mg/kg). It thus appears that there are some individuals with low expression of 3-FL, suggesting impaired ability to synthesize structures containing α-1,3-linked fucose. Low levels of 3-FL appear to be common in mothers with no expression of the Lewis genes [14,43,47,48]. However, even if those mothers typically have low levels of 3-FL in their milk, they are still higher (300–500 mg/L) than the very low levels (less than 43 mg/kg) observed in the milk of the 10 individuals in this study. Some previous studies [43,45,63] have also observed a few individuals with very low levels (0–50 mg/L) of 3-FL in their milk, similar to the levels observed here. Prieto [15] reported the incidence of two individuals out of a study of 435 in which 3-FL was absent in the milk. Large-scale studies such as the one reported here are required to pick up these unusual HMO profiles. Studying the genetic background of the individuals with unusual profiles could help us better understand how HMO synthesis is regulated; however it is not always foreseen (or possible) to collect such information

**Figure 2:**
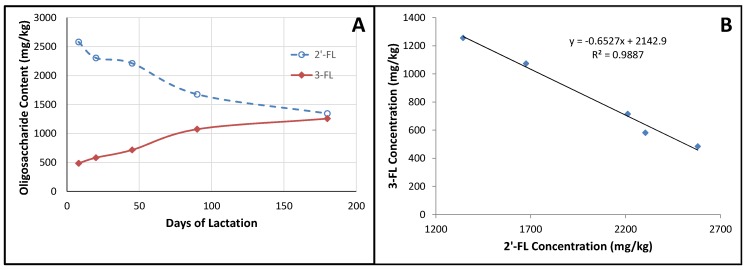
(**A**) Changes in 2′-FL and 3-FL concentration at different stages of lactation; (**B**) Correlation between 3-FL concentration and 2′-FL concentration.

**Figure 3:**
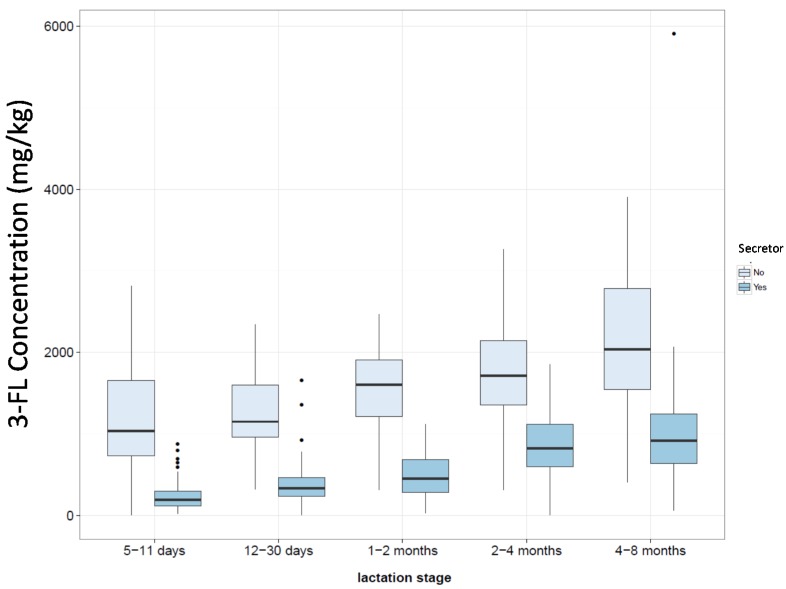
3-FL concentration in milk samples comparing secretor *vs.* non-secretor milk, where non-secretor status has been assigned to samples having 2′-FL content below the method LoQ.

When we were looking at how the oligosaccharide concentrations varied during lactation, we observed that 3-FL appeared to follow a similar trend to that of 2′-FL, but in the opposite direction (*i.e.*, when 2′-FL decreased, 3-FL increased; Figure 2A). When we plotted the mean concentration of 3-FL at each lactation stage against the mean concentration of 2′-FL at each lactation stage, it appeared that there was a strong correlation (Figure 2B). A correlation between these two oligosaccharides has previously been reported [64], although not as strong as the correlation observed here. Such a relationship suggests that the enzymes responsible for the synthesis of these oligosaccharides are either co-regulated or that they compete for a limited supply of the same substrate. Some clues may be obtained by comparing the milk of non-secretors against that of secretors. If the HMO production is fully regulated, one might expect the 3-FL level to be comparable between the two groups at the equivalent stage of lactation; on the other hand, if there is competition for a limited supply of substrate, then one would expect the non-secretor milks to have a higher 3-FL content than that of secretors. Considering the samples in this study with levels of 2′-FL below the LoQ to be non-secretors, such a comparison (Figure 3) shows that non-secretor milks tend to have a higher 3-FL content than that of secretor milks, suggesting that the relative levels of the fucosylated oligosaccharides are a result of the competition between the enzymes for a limited supply of substrate. Other studies [47,61] have also shown that non-secretors tend to have higher contents of 3-FL in their milk compared to secretors. Thus, we would speculate that the availability of guanosine 5′-diphosphate (GDP)-l-fucose may limit the total amount of fucosylated oligosaccharides present in the milk, while the expression levels and activities of the various fucosyltransferases control the relative contents of the different fucosylated structures.

One subject, out of the 446 studied, had levels of both 2′-FL and 3-FL below LoQ. In addition, levels of LNFP-V and LNnFP were below LoD, and the level of LNFP-I was only 19 mg/kg, the lowest above-LoQ level recorded in this study. This would imply that there are some members of the population in which either the availability of GDP-l-fucose for HMO synthesis is impaired or they have very poor expression levels of several fucosyltransferase enzymes. Unfortunately, within the 10 HMO studied, there are no oligosaccharides containing α-1,4-linked fucosyl residues, such as LNFP-II, which would have provided a deeper insight into what was happening in that particular individual. To the best of our knowledge, only one other large HMO study has reported a similar finding [14]. In that study, two samples were found that contained no 3-FL, one of which was devoid of both 3-FL and 2′-FL. The glycoproteins of the milk sample containing neither 2′-FL, nor 3-FL, were further analyzed and also found to be devoid of fucose, but it was not possible to investigate further to assess if the donor was unable to produce the GDP-l-fucose donor or if the donor did not express any functional fucosyltransferases.

### 4.3. Sialylated Oligosaccharides

3′-SL and 6′-SL were detected in all samples; the levels measured at equivalent time points were similar to those measured by Coppa [44] and Bao [54], but lower than those measured by Thurl [48]. 6′-SL is the predominant form at early stages of lactation; however, at later stages (beyond 2–4 months), the concentrations of the two sialyllactoses become similar, with the mean level of 3′-SL being higher at 4–8 months. In most previous studies, 6′-SL has been reported as the predominant form; however, only a few studies have gone beyond 1–2 months post-partum. The studies of Coppa [44] and Thurl [48] both measured sialyllactose levels in milk up to 90 days post-partum; in both cases, the 6′-SL remained the predominant form throughout. However, Sakaguchi [60] observed that 3′-SL became the predominant form of sialyllactose at three months post-partum, similar to what was observed in this study. Of all of the samples analyzed in this study, 36% had levels of 3′-SL greater than that of 6′-SL; in some individuals, the 3′-SL was present in concentrations up to four-times greater than that of 6′-SL. That difference could be greater in samples where 6′-SL concentrations were below the method LoQ.

### 4.4. LNT and LNnT

LNT and LNnT were present in all samples. Levels reported in the literature for these two oligosaccharides vary quite significantly. Galeotti [61] measured LNT levels as high as 5000 mg/L and LNnT as high as 4200 mg/L, and Nakhla [45] found levels of LNT and LNnT as low as 54 mg/L and 10 mg/L, respectively. The levels of LNT measured in this study, tend towards the central values of 700–1000 mg/L observed in other studies [44,45,50,51,52,55,58,59], while those for LNnT seem to be slightly lower than the central values of 250–700 mg/L. LNT and LNnT are two of the so-called core structures upon which many of the other HMO are based via additions of fucosyl- or sialyl-residues. They can also be elongated via additions of galactosyl and *N*-acetylglucosaminyl residues to produce other core structures, such as lacto-*N*-hexaose (LNH), lacto-*N*-octaose (LNO), *etc.* LNT is always the predominant of the two, which is a feature typical of, and unique to, human milk [5], in which type-I structures (those containing the Gal-β-1,3-GlcNAc moiety) are more abundant than type-II (containing the Gal-β-1,4-GlcNAc moiety). Both structures are probably used as substrates for growth by the gut microbiota, although type-I structures have been shown to be generally preferred by the Bifidobacteria [21], which contain the appropriate enzymatic machinery for their metabolism. Albrecht [19] and Coppa [20] compared the oligosaccharide composition of breast milk and of the feces of the babies fed that milk and demonstrated that the amount of oligosaccharides present in the feces was only about 40%–50% of that ingested, but the fecal levels of some oligosaccharides, including LNT, were reduced to a much greater extent than the other HMO. *In vitro* studies have suggested that LNT could provide protection against infection from the parasite Entamoeba histolytica by acting as a decoy structure for lectin binding [30].

## 5. Conclusions

A validated method has been established to measure the concentration of 10 oligosaccharides in human milk, including, for the first time, LNnFP and A-tetrasaccharide. The concentrations of the eight previously-studied oligosaccharides are in line with concentrations previously reported in different populations, and the fraction of the study population having low levels of α-1,2-linked fucosylated oligosaccharides (such as 2′-FL) is in line with the fraction of FUT2 non-secretors in other populations. However, in the Chinese population studied here, there is evidence that a low level of α-1,2-linked fucosylated oligosaccharides can always be synthesized.

Concentrations of 2′-FL, 3′-SL, 6′-SL, LNT, LNnT, LNFP-I and LNFP-V all decrease over the course of lactation, while the concentration of 3-FL increases. Concentrations of 2′-FL and 3-FL are strongly correlated with one another, implying some sort of mechanism for co-regulation. 6′-SL is generally considered to be the predominant of the two sialyllactoses in human milk. This is true at early stages of lactation; however, in this study, 3′-SL became the predominant form beyond 2–4 months post-partum. Concentrations of the oligosaccharides changed with stage of lactation, but were not correlated with geographical location within China, nor mode of delivery. It is well established that the HMO profile is influenced by genetics and by the stage of lactation. We have shown that the mode of delivery does not seem to influence the HMO profile, but questions remain as to whether other environmental factors, such as diet, may have some influence.

## Figures and Tables

**Table 1:** Study population characteristics.

*n*	5–11 Days	12–30 Days	1–2 Months	2–4 Months	4–8 Months
	90	90	90	90	90
**Mothers**					
**Age (years), mean (SD)**	27 (4)	27 (3)	28 (4)	27 (4)	26 (4)
**Height (m), mean (SD)**	160 (4)	160 (5)	161 (5)	161 (5)	159 (5)
**Weight (kg), mean (SD)**	60.7 (8.7)	60.8 (7.9)	61.9 (8.9)	58.4 (8.3)	56.2 (8.1)
**BMI (kg/m^2^), mean**	23.7 (3.2)	23.7 (3.0)	23.9 (3.1)	22.5 (2.9)	22.2 (3.1)
**Gestational weight gain (kg), mean (SD)**	16.7 (7.4)	16.2 (6.0)	15.9 (5.7)	15.9 (5.9)	14.9 (7.6)
**Postpartum weight loss (kg), mean (SD)**	9.1 (6.1)	8.6 (5.3)	9.8 (4.0)	10.0 (6.2)	10.6 (5.9)
**Non-smokers (%)**	100	99	100	98	100
**Cesarean delivery (%)**	42	48	59	39	38
**Household income (RMB/Month)**					
**<2000**	22	19	27	29	34
**2000–4000**	41	50	46	44	46
>**4000**	33	24	26	24	20
**Unknown**	1	7	2	0	0
**Infant**					
**Males (%)**	57	53	53	60	48
**Gestational age at birth (weeks), mean (SD)**	39.3 (1.2)	39.2 (1.3)	39.2 (1.6)	39.4 (1.3)	39.5 (1.5)

**Table 2:** Analytical method performance.

HMO	Linear Range	LoD	LoQ	RSD_(r)_^a^	RSD_(iR)_^a^	Native Content	High Spike	Medium Spike	Low Spike	Spike Recovery ^c^ (%)		
	(mg/kg)	(mg/kg)	(mg/kg)	(%)	(%)	(mg/kg)	(mg/kg)	(mg/kg)	(mg/kg)	High	Med	Low
2′-FL	52.76–5276	4.4	53	1.8	3.2	1100	586	293	58.6	100	100	105
3-FL	43.33–4333	3.6	43	4.4	4.7	785	481	241	48.1	99.7	99.6	101
3′-SL	23.01–2301	1.9	23	2.7	3.6	79.5	256	128	25.6	101	99.5	101
6′-SL	22.21–2221	1.9	22	1.8 ^b^	2.6 ^b^	20.0	247	123	24.7	101	99.9	97.4
A-Tetra	19.58–1958	4.9	20	2.4 ^b^	2.8 ^b^	nd ^d^	218	109	21.8	99.9	98.7	100
LNT	14.00–5600	4.7	14	1.7	3.8	382	622	311	62.2	100	98.8	104
LNnT	19.96–1996	5.0	20	1.6	4.7	93.3	222	111	22.2	100	100	101
LNFP-I	14.95–5979	5.0	15	2.1	3.6	167	664	332	66.4	100	99.0	103
LNFP-V	13.36–1336	3.3	13	8.1	8.1	20.1	148	74.2	14.8	101	102	105
LNnFP	12.42–1241	3.1	12	2.1 ^b^	2.8 ^b^	8.85	138	69.0	13.8	100	99.6	101

^a^ RSD_(r)_ and RSD_(iR)_ were all determined by analyzing a non-spiked sample on 6 different days in duplicate, except for ^b^, which were determined on the low spike sample since the milk sample being used for validation did not contain HMO levels above the LoQ; ^c^ a milk sample was spiked at three different levels and analyzed on 6 different days in duplicate; the reported recoveries are the average; ^d^ nd = not detected.

**Table 3:** Concentration of oligosaccharides in human milk, with samples having concentrations >LoD, but <LoQ assigned a value of half the LoQ.

Oligosaccharide		Lactation Stage	*n* > LoD	*n* < LoD	Oligosaccharide Concentration (mg/kg)				
					Min	Max	Mean	Median	SD
2′-FL	α-l-Fuc-(1→2)-β-d-Gal-(1→4)-d-Glc	5–11 days	88	0	26	4900	2000	2100	1400
		12–30 days	88	0	26	4000	1900	1900	1200
		1–2 months	90	0	26	4400	1700	1800	1100
		2–4 months	90	0	26	3800	1300	1300	900
		4–8 months	90	0	26	3000	1100	1200	710
3-FL	β-d-Gal-(1→4)-[α-l-Fuc-(1→3)-]-d-Glc	5–11 days	88	0	22	2800	490	230	600
		12–30 days	88	0	22	2400	570 ^a,b^	400	480
		1–2 months	90	0	22	2500	720 ^b^	620	550
		2–4 months	90	0	22	3300	1100 ^a,b^	980	610
		4–8 months	90	0	63	5900	1300 ^b^	1100	900
3′-SL	α-d-Neu5Ac-(2→3)-β-d-Gal-(1→4)-d-Glc	5–11 days	88	0	60	230	110	110	35
		12–30 days	88	0	53	180	94 ^a,b^	87	25
		1–2 months	90	0	49	160	80 ^a,b^	77	22
		2–4 months	90	0	43	140	79 ^b^	75	20
		4–8 months	90	0	46	260	83 ^b^	77	28
6′-SL	α-d-Neu5Ac-(2→6)-β-d-Gal-(1→4)-d-Glc	5–11 days	88	0	11	690	330	340	140
		12–30 days	88	0	68	600	250 ^a,b^	250	93
		1–2 months	90	0	32	420	140 ^a,b^	120	81
		2–4 months	90	0	25	210	78 ^a,b^	70	40
		4–8 months	90	0	11	99	39 ^a,b^	35	21
A-Tetra	α-d-GalNAc-(1→3)-[α-l-Fuc-(1→2)]-β-d-Gal-(1→4)-d-Glc	5–11 days	18	70	9.8	47	13	9.8	9.5
		12–30 days	13	75	9.8	160	27	9.8	42
		1–2 months	12	78	9.8	140	25	9.8	36
		2–4 months	13	77	9.8	57	18	9.8	15
		4–8 months	9	81	9.8	68	22	9.8	21
LNT	β-d-Gal-(1→3)-β-d-GlcNAc-(1→3)-β-d-Gal-(1→4)-d-Glc	5–11 days	88	0	97	3000	880	790	530
		12–30 days	88	0	130	2200	620 ^a,b^	550	340
		1–2 months	90	0	95	1200	370 ^a,b^	290	220
		2–4 months	90	0	41	960	290 ^a,b^	250	170
		4–8 months	90	0	21	750	250 ^b^	190	160
LNnT	β-d-Gal-(1→4)-β-d-GlcNAc-(1→3)-β-d-Gal-(1→4)-d-Glc	5–11 days	88	0	10	390	180	170	85
		12–30 days	88	0	23	370	120 ^a,b^	110	67
		1–2 months	90	0	10	240	83 ^a,b^	81	43
		2–4 months	90	0	10	170	65 ^a,b^	54	39
		4–8 months	90	0	10	200	50 ^a,b^	43	36
LNFP-I	α-l-Fuc-(1→2)-β-d-Gal-(1→3)-β-d-GlcNAc-(1→3)-β-d-Gal-(1→4)-d-Glc	5–11 days	81	7	7.5	4000	910	880	740
		12–30 days	80	8	7.5	1700	540	490	400
		1–2 months	74	16	7.5	1400	340 ^b^	290	240
		2–4 months	73	17	7.5	660	180 ^a,b^	140	140
		4–8 months	74	16	7.5	860	160 ^b^	110	150
LNFP-V	β-d-Gal-(1→3)-β-d-GlcNAc-(1→3)-β-d-Gal-(1→4)-[α-l-Fuc-(1→3)-]-d-Glc	5–11 days	86	2	6.7	250	41	22	49
		12–30 days	84	4	6.7	240	39	24	41
		1–2 months	86	4	6.7	110	26 ^a,b^	16	25
		2–4 months	87	3	6.7	130	25	19	24
		4–8 months	88	2	6.7	75	20 ^b^	18	15
LNnFP	β-d-Gal-(1→4)-β-d-GlcNAc-(1→3)-β-d-Gal-(1→4)-[α-l-Fuc-(1→3)-]-d-Glc	5–11 days	80	8	6.2	51	11	6.2	9.0
		12–30 days	83	5	6.2	31	8.1 ^a,b^	6.2	4.7
		1–2 months	81	9	6.2	22	7.9 ^b^	6.2	4.1
		2–4 months	79	11	6.2	21	8.2 ^b^	6.2	4.1
		4–8 months	75	15	6.2	23	7.5 ^b^	6.2	3.7

**^a^** The contrast estimate indicates that the HMO concentration at that time point is significantly different (*p* < 0.05) from the concentration of the immediately preceding time point; ^b^ the contrast estimate indicates that the HMO concentration at that time point is significantly different (*p* < 0.05) from the concentration at 5 to 11 days.

**Table 4:** Concentration of oligosaccharides in human milk, with samples having concentrations <LoQ removed from the dataset.

Oligosaccharide		Lactation Stage	*n* > LoQ	*n* < LoQ	Oligosaccharide Concentration (mg/kg)				
					Min	Max	Mean	Median	SD
2′-FL	α-l-Fuc-(1→2)-β-d-Gal-(1→4)-d-Glc	5–11 days	67	21	56	4900	2600	2500	970
		12–30 days	70	18	290	4000	2300	2300	800
		1–2 months	71	19	690	4400	2200	2100	730
		2–4 months	71	19	84	3800	1800 ^a,b^	1600	670
		4–8 months	71	19	290	3000	1300 ^a,b^	1400	510
3-FL	β-d-Gal-(1→4)-[α-l-Fuc-(1→3)-]-d-Glc	5–11 days	84	4	47	2800	510	250	600
		12–30 days	86	2	50	2300	580 ^a,b^	400	480
		1–2 months	88	2	63	2500	730 ^b^	620	550
		2–4 months	88	2	72	3300	1100 ^a,b^	990	600
		4–8 months	90	0	63	5900	1300 ^b^	1100	900
3′-SL	α-d-Neu5Ac-(2→3)-β-d-Gal-(1→4)-d-Glc	5–11 days	88	0	60	230	110	110	350
		12–30 days	88	0	53	180	94 ^a,b^	87	25
		1–2 months	90	0	49	160	80 ^a,b^	77	22
		2–4 months	90	0	43	140	79 ^b^	75	20
		4–8 months	90	0	46	260	83 ^b^	77	28
6′-SL	α-d-Neu5Ac-(2→6)-β-d-Gal-(1→4)-d-Glc	5–11 days	85	3	33	690	340	350	120
		12–30 days	88	0	68	600	250 ^a,b^	250	93
		1–2 months	90	0	32	420	140 ^a,b^	120	81
		2–4 months	90	0	25	210	78 ^a,b^	70	40
		4–8 months	73	17	23	99	45 ^a,b^	42	18
A-Tetra	α-d-GalNAc-(1→3)-[α-l-Fuc-(1→2)]-β-d-Gal-(1→4)-d-Glc	5–11 days	2	86	28	47	38	380	13
		12–30 days	3	85	31	160	86	71	64
		1–2 months	4	86	22	140	56	32	54
		2–4 months	4	86	20	57	36	33	17
		4–8 months	3	87	34	68	46	37	19
LNT	β-d-Gal-(1→3)-β-d-GlcNAc-(1→3)-β-d-Gal-(1→4)-d-Glc	5–11 days	88	0	97	3000	880	790	530
		12–30 days	88	0	130	2200	620 ^a,b^	550	340
		1–2 months	90	0	95	1200	370 ^a,b^	290	220
		2–4 months	90	0	41	960	290 ^a,b^	250	170
		4–8 months	90	0	21	750	250 ^b^	190	160
LNnT	β-d-Gal-(1→4)-β-d-GlcNAc-(1→3)-β-d-Gal-(1→4)-d-Glc	5–11 days	85	3	26	390	180	170	81
		12–30 days	83	5	23	370	120 ^a,b^	110	67
		1–2 months	87	3	23	240	85 ^a,b^	82	42
		2–4 months	83	7	20	170	69 ^a,b^	59	38
		4–8 months	74	16	21	200	59 ^a,b^	49	34
LNFP-I	α-l-Fuc-(1→2)-β-d-Gal-(1→3)-β-d-GlcNAc-(1→3)-β-d-Gal-(1→4)-d-Glc	5–11 days	68	20	15	4000	1100	1000	690
		12–30 days	71	17	39	1700	600	530	370
		1–2 months	73	17	20	1400	340	290	240
		2–4 months	70	20	21	660	190 ^a,b^	150	140
		4–8 months	69	21	21	860	170 ^b^	120	150
LNFP-V	β-d-Gal-(1→3)-β-d-GlcNAc-(1→3)-β-d-Gal-(1→4)-[α-l-Fuc-(1→3)-]-d-Glc	5–11 days	58	30	14	250	57	30	53
		12–30 days	64	24	13	240	49	34	42
		1–2 months	52	38	14	110	38 ^a,b^	26	25
		2–4 months	57	33	14	130	35	26	24
		4–8 months	54	36	14	75	29 ^b^	24	13
LNnFP	β-d-Gal-(1→4)-β-d-GlcNAc-(1→3)-β-d-Gal-(1→4)-[α-l-Fuc-(1→3)-]-d-Glc	5–11 days	24	62	13	51	22	18	9.9
		12–30 days	14	74	13	31	17 ^a,b^	16	5.3
		1–2 months	13	77	13	22	17	17	3.4
		2–4 months	17	73	13	21	16	14	3.1
		4–8 months	9	81	13	23	17 ^b^	17	3.4

^a^ The contrast estimate indicates that the HMO concentration at that time point is significantly different (*p* < 0.05) from the concentration of the immediately preceding time point; ^b^ the contrast estimate indicates that the HMO concentration at that time point is significantly different (*p* < 0.05) from the concentration at 5 to 11 days.

## Abbreviations

The following abbreviations are used in this manuscript:

2′-FL	2′-fucosyllactose
3-FL	3-fucosyllactose
3′-SL	3′-sialyllactose
6′-SL	6′-sialyllactose
A-Tetra	A-tetrasaccharide
CE	Capillary electrophoresis
Fuc	Fucose
FucT2	Fucosyltransferase-2
Gal	Galactose
GDP	Guanosine 5′-diphosphate
Glc	Glucose
GlcNAc	*N*-acetylglucosamine
HMO	Human milk oligosaccharides
HPLC	High performance liquid chromatography
LNFP-I	Lacto-*N*-fucosylpentaose-I
LNFP-II	Lacto-*N*-fucosylpentaose-II
LNFP-III	Lacto-*N*-fucosylpentaose-III
LNFP-V	Lacto-*N*-fucosylpentaose-V
LNH	Lacto-*N*-hexaose
LNnFP	Lacto-*N*-neofucosylpentaose
LNnT	Lacto-*N*-neotetraose
LNO	Lacto-*N*-octaose
LNT	Lacto-*N*-tetraose
LoD	Limit of detection
LoQ	Limit of quantification
MS	Mass spectrometry
Neu5Ac	*N*-acetylneuraminic acid
NLO	Non-lactose oligosaccharide
NMR	Nuclear magnetic resonance spectroscopy
PAD	Pulsed amperometric detection
UV	Ultraviolet detection
